# In-silico engineering of RNA nanoplatforms to promote the diabetic wound healing

**DOI:** 10.1186/s13065-023-00969-4

**Published:** 2023-06-08

**Authors:** Nima Beheshtizadeh, Alireza Salimi, Mahsa Golmohammadi, Javad Mohajer Ansari, Mahmoud Azami

**Affiliations:** 1 Department of Tissue Engineering, School of Advanced Technologies in Medicine, Tehran University of Medical Sciences, Tehran, Iran; 2 Regenerative Medicine group (REMED), Universal Scientific Education and Research Network (USERN), Tehran, Iran; 3 Students? Scientific Research Center, Tehran University of Medical Sciences, Tehran, Iran; 4 Department of Advanced Technologies, School of Medicine, North Khorasan University of Medical Science, Bojnurd, Iran; 5 Department of Polymer Engineering and Color Technology, Amirkabir University of Technology, Tehran, Iran; 6 Department of Anatomy, School of Medicine, Hormozgan University of Medical Sciences, Jomhuri Eslami Blvd, Bandar Abbas, 7919915519 Iran

**Keywords:** RNA interferences, Diabetic foot ulcers, Wound healing, Angiogenesis, microRNA, Tissue engineering

## Abstract

One of the most notable required features of wound healing is the enhancement of angiogenesis, which aids in the acceleration of regeneration. Poor angiogenesis during diabetic wound healing is linked to a shortage of pro-angiogenic or an increase in anti-angiogenic factors. As a result, a potential treatment method is to increase angiogenesis promoters and decrease suppressors. Incorporating microRNAs (miRNAs) and small interfering RNAs (siRNAs), two forms of quite small RNA molecules, is one way to make use of RNA interference. Several different types of antagomirs and siRNAs are now in the works to counteract the negative effects of miRNAs. The purpose of this research is to locate novel antagonists for miRNAs and siRNAs that target multiple genes to promote angiogenesis and wound healing in diabetic ulcers.

In this context, we used gene ontology analysis by exploring across several datasets. Following data analysis, it was processed using a systems biology approach. The feasibility of incorporating the proposed siRNAs and miRNA antagomirs into polymeric bioresponsive nanocarriers for wound delivery was further investigated by means of a molecular dynamics (MD) simulation study. Among the three nanocarriers tested (Poly (lactic-co-glycolic acid) (PLGA), Polyethylenimine (PEI), and Chitosan (CTS), MD simulations show that the integration of PLGA/hsa-mir-422a is the most stable (total energy = -1202.62 KJ/mol, Gyration radius = 2.154 nm, and solvent-accessible surface area = 408.416 nm^2^). With values of -25.437 KJ/mol, 0.047 nm for the Gyration radius, and 204.563 nm^2^ for the SASA, the integration of the second siRNA/ Chitosan took the last place. The results of the systems biology and MD simulations show that the suggested RNA may be delivered through bioresponsive nanocarriers to speed up wound healing by boosting angiogenesis.

**Supplementary Information:**

The online version contains supplementary material available at 10.1186/s13065-023-00969-4.

## Introduction

Approximately 15–25% of type 2 diabetic patients suffer from diabetic foot ulcers [1]. Destruction of the integumentary system (often dermis and epidermis) and subcutaneous tissue occurs in diabetic ulcers in multiple cases, which lead to amputation in 14–24% of affected patients [2]. As a result of diabetic foot ulcers, lower-extremity amputation is a high-risk procedure with a high percentage of five-year mortality (50–59%), higher than many cancers [3]. Due to the massive complications followed by diabetic ulcers, acceleration and improvement in diabetic ulcers healing is a research priority.

Enhancement of angiogenesis, which contributes to wound healing acceleration, is one of the distinguishing characteristics of tissue regeneration. Angiogenesis is regulated by multiple biochemical factors, including hypoxia-inducible-factor-1 (HIF-1), vascular-endothelial-growth-factor (VEGF), transforming-growth-factor (TGF), and fibroblast-growth-factors (FGF), and results in the development of new arteries [4]. Inflammation, proliferation, and remodeling are the three stages of the wound healing process. When skin is damaged, inflammatory cells are driven to the site of the lesion, which is followed by fibroblast growth, and these cells are responsible for generating numerous tissue components [5]. Normal wound healing requires angiogenesis stimulation; however, in diabetic wounds, hyperglycemia as a condition inhibits angiogenesis and transactivates hypoxia-inducible factor (HIF) [6]. A capillary bed is developed in the wound area regarding angiogenic factors, during the proliferative phase of the wound healing. Interestingly, diabetes increases angiogenesis in diabetic retinopathy or nephropathy, although it declines angiogenesis in wound healing [7]. Poor angiogenesis process during diabetic wound healing is associated either with insufficiency in pro-angiogenic or an increase in anti-angiogenic factors [8]. Therefore, improvement of promoters or hallmarks of suppressors of angiogenesis could be used as a therapeutic strategy.

TGF-1 is a versatile cytokine that promotes granulation tissue development and collagen production during wound healing [9]. Polyamines increased the expression of ODC, SSAT, hypoxia-inducible factors-1 (HIF-1), VEGF, and matrix metalloproteinases (MMOs) while decreasing the expression of p27 in Akt1-overexpression cells [10]. The proliferation, differentiation, and migration of multiple cell types are all regulated by FGF2, a growth factor whose expression is decreased in diabetic and pressure ulcer tissues. For instance, wound healing, angiogenesis, bone regeneration and neuroregeneration may all benefit from FGF2’s participation in the formation of new blood vessels and tissue regeneration [11].

Ribonucleic acid interference (RNAi) is a ubiquitous biological response to double-stranded RNA that offers resistance to endogenous parasites and external harmful nucleic acids and controls protein-coding gene expression. Knocking out or down the genes is associated with numerous biological processes and is regarded as a crucial method in molecular research [12]. The RNAi technology may help accomplish this goal. This naturally occurring technique to sequence-specific gene silencing has the potential to advance experimental biology. It has potential applications in functional genomics, therapeutic intervention, regenerative medicine, and other areas.

RNAi strategy is based on two types of small RNA molecules: microRNA (miRNA) and small interfering RNA (siRNA) [13]. miRNAs are single strands (18–23 nucleotides), small non-coding, and endogenous products which bind to targeted miRNAs and degrade or block the translation of genes [13]. Hence, miRNAs might have significant effects on several pathological and physiological processes. It has been shown that miRNAs upregulate and downregulate those genes associated with angiogenesis, cell proliferation, and collagen synthesis during the wound healing process [14]. Due to the therapeutic potential, the application of anti-miRNAs or antagomirs may inhibit miRNAs’ undesirable effects, including angiogenesis suppression [15].

The primary function of miRNAs is to regulate gene expression. A miRNA is an RNA that is complementary to a portion of one or more mRNAs. The sequence in the 5′ proximal region between the 2nd and 8th nucleotides is where miRNAs mainly interact with their mRNA targets. It is worth noting that miRNA interacts with expression of target genes by repressing mRNA translation or degrading mRNA through deadenylation from the 3′ end and/or decapping from the 5′ end. According to a recent research, translation suppression regulates 48% of miRNA target genes, mRNA degradation influences 29%, and combined mechanisms control 23% [16].

Along with miRNAs, siRNAs play a significant role in the RNAi process leading to the knockdown of target genes. The process of gene silencing conducted by siRNAs is more specific than by miRNAs, whereas miRNAs usually affect multiple target genes [17]. Synthetic siRNAs have emerged as prospective therapeutic agents for multiple issues, such as cancer, metabolic disorders, inflammatory disorders, and infectious diseases [18]. Due to the different approaches, the application of siRNAs and miRNAs in pharmaceutical subjects may be considered a combined or parallel therapy.

Multiple therapeutic approaches to siRNAs have been developed and gained momentum in the past years [19, 20]. In the context of diabetic wound healing, siRNAs are designed to minimize undesired effects on healing processes through downregulation of genes such as P53 (increase apoptosis and impair vasculogenesis), matrix metalloproteinase (MMP9) (deteriorate of extracellular matrix), prolyl-hydroxylase domain 2 (PHD-2) (destabilize HIF-1) [21–23]. Therefore, utilizing siRNAs could improve and hasten cure for various phases of tissue regeneration and wound healing.

In terms of improving angiogenesis and tissue regeneration in diabetic foot ulcers, multiple miRNAs have been investigated. Diabetes foot ulcer lesions were shown to include extracellular vesicles expressing miR-195-5p and miR-205-5p, which inhibit angiogenesis and tissue regeneration in diabetes foot patients [24]. In both in vivo and in vitro testing, the use of miRNA-497 as a therapy resulted in a reduction of pro-inflammatory cytokines such as IL-1, IL-6, and TNF-. The anti-inflammatory effects of miRNA-497 provide new information on the role it plays in facilitating the speedy recovery of diabetic wounds. As a result of its pro-inflammatory cytokine down-regulation capabilities, miRNA-497 has been indicated as a viable therapy option for diabetic wound healing [25].

The antiangiogenic microRNA 92a-3p (miR-92a) has been shown to increase angiogenesis in a variety of organ systems, including the heart, the limbs, the blood vessels, and the bones [26]. It has been shown that a synthetic miR-92a inhibitor (MRG-110) can increase ITGA5 expression in vitro in both human vascular endothelial cells and primary human skin fibroblasts, and in mice skin [26]. According to these results, MRG-110 has the potential to improve contextual chronic wound healing as well as acute wound healing [27].

On the other hand, the basis of biological activity is molecular interactions [28], which are the product of macromolecular structures [29]. Simulations of molecular dynamics (MD) have grown into a sophisticated approach for determining the links between macromolecular structure and function [30]. Also, physiologically-relevant periods may be compared to the simulation process timeframes. For structural bioinformatics, the knowledge on dynamic macromolecule features is sufficient to transform the paradigm from single-structure studies to conformational ensemble evaluation [30]. MD simulation, capable of modeling the biological macromolecules such as proteins and genes, and their integration with various materials, including polymeric nanocarriers, discusses potential therapeutic approaches for multiple diseases.

**Fig. 1:**
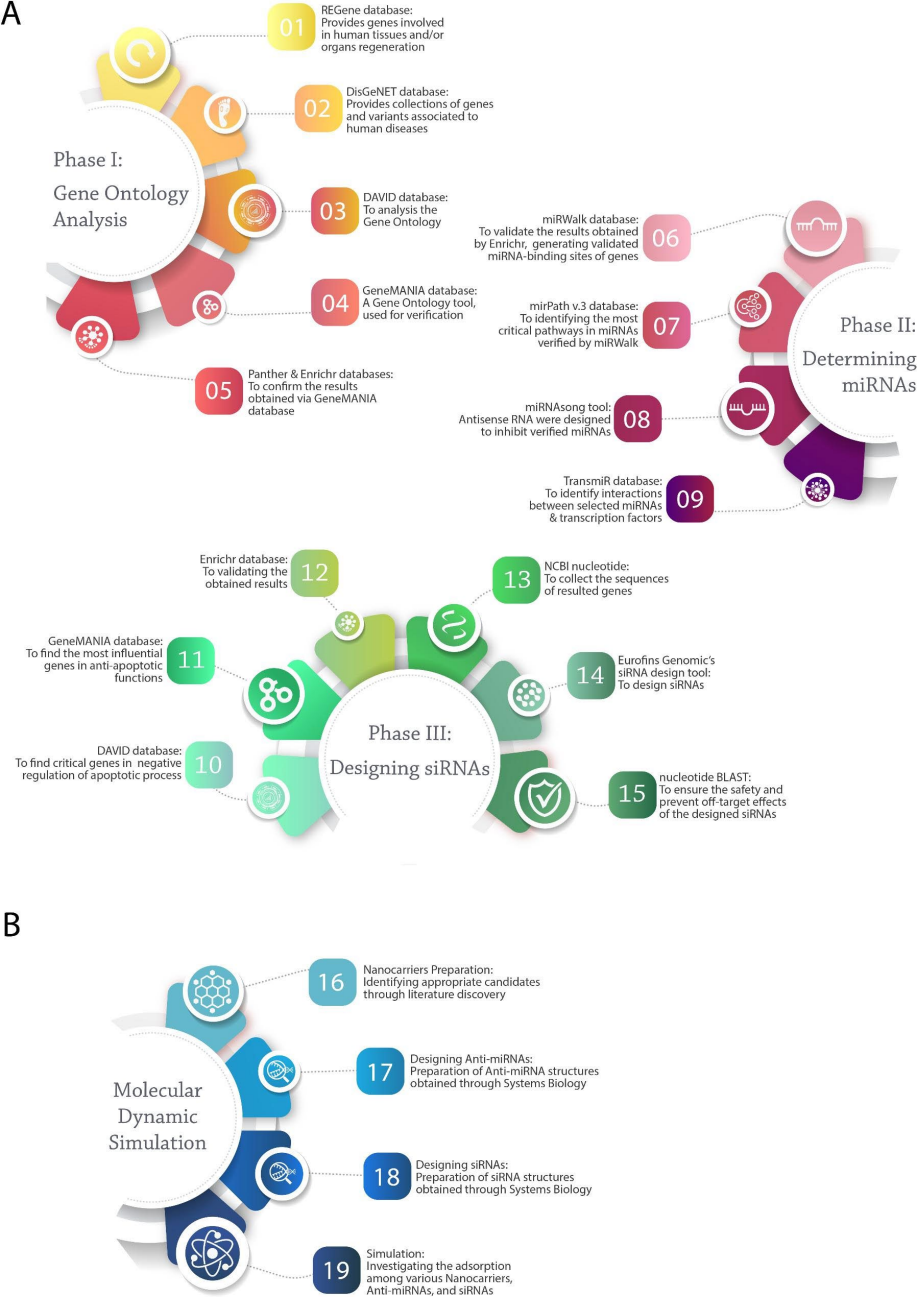
Study procedures consist of 19 various steps: (A) Systems biology evaluation was performed in three phases, including Gene Ontology analysis, determining miRNAs, and designing siRNAs, from steps 1–15. (B) Molecular dynamic simulation is performed in 4 steps

To date, multiple antagomirs and siRNAs have been presented to reduce the undesirable effects of miRNAs. To the best of our knowledge, there is not a study that could suggest antagomirs and siRNAs, and discover their delivery via employing bioresponsive nanocarriers at the wound site. Almost previous studies lack the survey of employment possibility of designed and dedicated small molecules in diabetic wound healing. This paper aims to present new antagomirs for miRNAs and siRNAs, which suppress the anti-angiogenesis process by targeting multiple genes and improving the wound-healing process. For this object, the gene ontology algorithms were used by utilizing several databases. Then, a systems biology approach was applied to the whole gathered data. Also, to deliver the designed siRNAs and miRNAs’ antagomirs to the wound site, their integration with three types of various polymeric nanocarriers was considered through an MD simulation study. Figure 1 summarizes the whole study procedure.

Figure 1.

## Materials and methods

### Gene ontology analysis

A systems biology investigation was carried out in this paper, which made use of the regeneration gene database as well as the DisGeNET database (v7.0). All procedures were carried out in conformity with the applicable guidelines and regulations. The regeneration gene database contains genes involved in the regeneration of human tissues and/or organs, while DisGeNET is a discovery platform that contains one of the biggest publicly accessible collections of genes and variations related with human disorders [31, 32].

Using the database for annotation, visualization and integrated discovery (DAVID) database, each of the obtained genes was subjected to an analysis designed to enrich individual gene ontology (GO) terms in order to determine whether or not co-transcriptionally regulated genes share any activities related to wound healing [33, 34]. DAVID distinguishes itself from comparable databases by providing an integrated and enlarged back-end annotation database, innovative modular enrichment techniques, and strong exploratory capability in an integrated data-mining context. One of the most critical elements that directly influences the performance of DAVID’s functional analysis is the quality of massive gene lists obtained from high-throughput biological investigations [35].

The genes resulted from DAVID entered in another online tool, enriching for GO terms, which is called GeneMANIA [36] to identify potential partners for angiogenesis, epithelial cell proliferation, regulation of epithelial cell migration, response to fibroblast growth factor, fibroblast migration, epithelial cell migration, and positive regulation of cell migration. A list of genes was loaded into GeneMANIA, and a list of data sets to query was chosen. GeneMANIA then expands the list with functionally related genes or genes that share attributes with the original query genes, displaying a dynamic functional correlation matrix that illustrates the links between the genes and datasets. Another notable feature of GeneMANIA is that it applies weights to data sets depending on their utility for each query [37].

To confirm the results obtained via the GeneMANIA database, pathway analysis and GO analysis were accomplished using the protein analysis through evolutionary relationships (Panther) [38] and Enrichr [39, 40] databases. The Panther categorization process is a systematic maintained biological database of gene/protein families and functionally related subfamilies that may be used to categorize and identify gene products’ functions. Panther’s practical application is to correctly estimate the function of uncharacterized genes from any organism based on evolutionary links to genes with known functions [41]. Enrichr, a web-based gadget that provides a variety of graphical summaries of the aggregate functions of gene lists, is very straightforward to use [39].

### Determining appropriate miRNAs

Enrichr database identified miRNAs, which potentially could target provided genes via GeneMANIA and Panther. To confirm the Enrichr database findings, they were further investigated using the miRWalk database [42], which is an open-source platform with an uncomplicated interface for producing predicted and verified miRNA-binding sites of known genes in humans, mice, rats, dogs, and cows [42]. Verified miRNAs were entered into the mirPath v.3 database to identify the most critical pathways, interfered with by these miRNAs and have a crucial role in wound healing [43].

To inhibit verified miRNAs, antisense RNAs were designed utilizing the miRNAsong tool [44]. miRNAsong is a web-based application for creating and testing miRNA sponges in a virtual environment. miRNAsong creates miRNA sponge constructs for certain miRNAs and miRNA families or clusters and then tests these constructs to see whether they have the ability to bind to miRNAs in a variety of species [44]. Finally, to identify interactions between selected miRNAs and TFs, the transmiR database was used [45, 46]. It is a significant resource for investigating TF–miRNA regulation and could be used to evaluate a wide range of processes, such as the development of the relationships, expression patterns, and related disorders of miRNAs [47].

### siRNA designing

The DAVID database was utilized to identify essential genes in the negative regulation of the apoptotic process. The findings were examined using the GeneMANIA database to identify the most significant genes in anti-apoptotic activities such as apoptotic signaling pathway negative regulation, intrinsic apoptotic signaling pathway negative regulation, and endothelial cell apoptotic process negative regulation. The Enrichr database was utilized to verify the acquired findings.

In this regard, the sequences of NFE2L2 and SIRT1 genes were collected from NCBI nucleotide [48]. To design siRNAs, Eurofins Genomic’s siRNA design tool was used, which is an online gadget providing the use of guidelines initially provided by Tuschl et al. [49]. In addition, it applies the scorings criteria [12] (including target site, length of siRNA, specificity checking, and nucleotide content of siRNA) developed by Reynolds et al. [50], Ui-Tei et al. [51], and Vert et al. [52]. To ensure the safety and prevent off-target effects of the designed siRNAs, the sequences were aligned by the human genome utilizing align sequences nucleotide BLAST [53].

### Molecular dynamics simulations

An MD simulation was performed using the all-atom technique (AA-MD) considering the PLGA, PEI and Chitosan nanocarriers, utilizing the OPLSA force field. Mentioned nanocarriers were designed using the Pepdraw and rcsb online tools and were parameterized with the gmx pdb2 gmx command in GROMAX 2020. Parameters like length, mass, net charge, and hydrophobicity were also provided utilizing Pepdraw tool and molecular dynamics add-ons, such as Avogadro. Polymeric nanocarriers are also designed by Avogadro software, then the parameter is pooled using PolyParGen online tool, while their esp charge were calculated by cp2k software. 18 simulations were performed with a duration of 120 nanoseconds and a time step of 2 femtoseconds. The simulations are then performed at the temperature and pH of 30.91 °C and 6.95, respectively [54]. All the simulation was performed in 4 steps using the coordinate file of the molecules. Initially, the simulation system was placed at an energy level of 100 (kJ/mol) after 100 ps. Next, using the Berendsen thermostat algorithm, the simulation system was placed at 30.91 °C after 1 ns. After the NVT (constant number of atoms, N; constant volume, V; constant temperature, T) stage, using the Parrinello-rahman algorithm and the simulation system equilibrates after 10 ns at a pressure of 1 bar. Finally, the simulation was performed using the LINCS algorithm at 50 ns, the cutoff radius was set to 3 nm and H-bonds were considered for all simulations. The tip3p water model was used for simulation. The simulations are performed with 1080 ti graphics. For the analyzes, the commands gmx gyrate, mmpbsa, gmx sasa were used, and for imaging the simulation system, vmd software was used. For molecular dynamics, GROMACS 2020 was used. Gmx gyrate calculate the gyration radius during the simulations using Eq. (1):

1 $${R}_{g}=\sqrt{\frac{{\sum }_{\dot{i}=1}^{n}{r}_{i}^{2}{m}_{i}}{{\sum }_{i=1}^{n}{m}_{i}}}$$

where n is the number of particles, mi, the mass of the particle i, and r_i_ indicate the distance of the particle i from the center of gravity. R_g_, helps to understand protein density during the simulation, where for higher R_g_, higher density is expected. Similarly, gmx sasa calculates the solvent accessible surface area of the particles in the course of simulations, via the double cubic lattice method [55].

## Results and discussion

### Gene ontology analysis and RNAis proposing

**Fig. 2:**
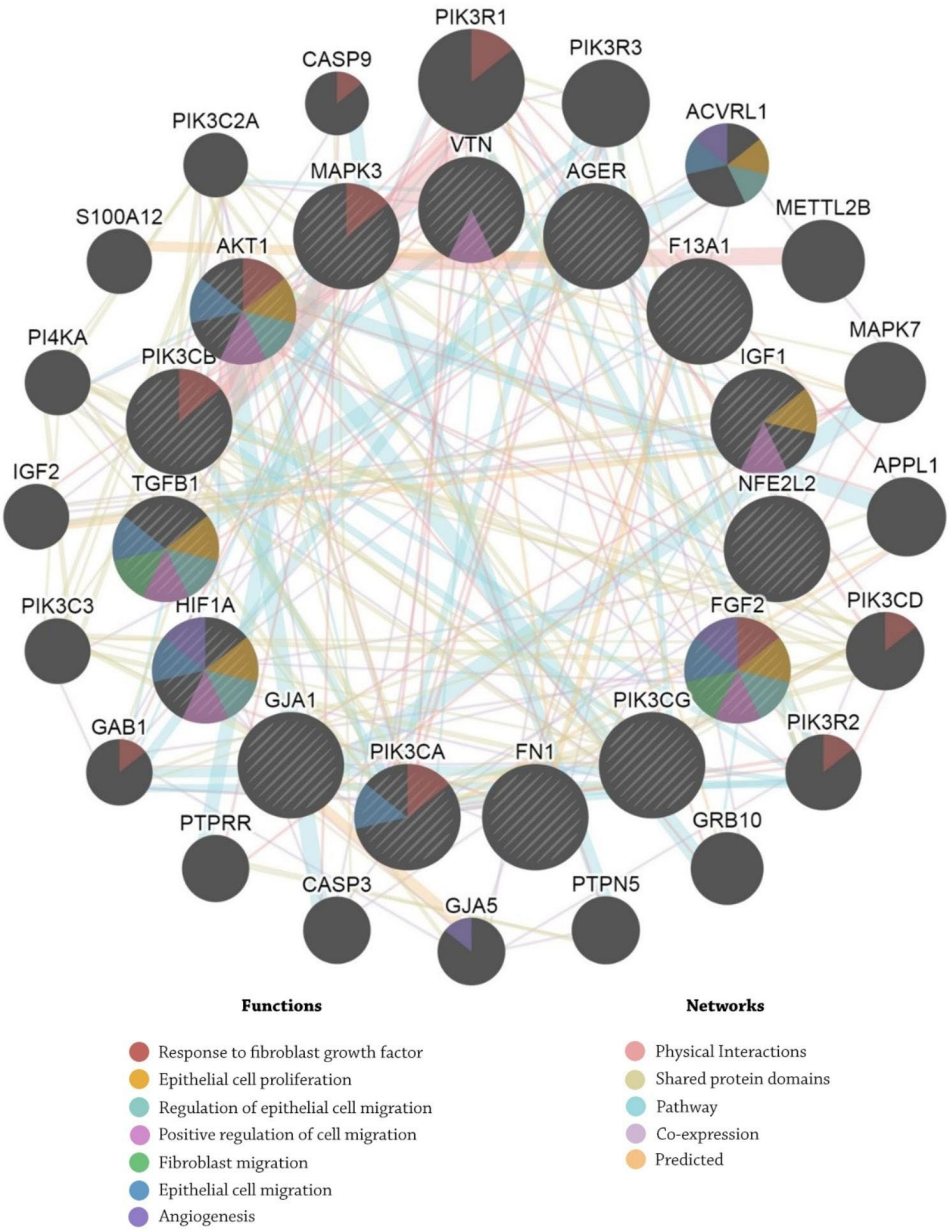
Analysis in the GeneMANIA database shows that six genes collaborate with each other in angiogenesis and proliferation processes, including AKT1, HIF1A, FGF2, IGF1, TGFB1, and PIK3CA.

All 72 genes that are participated in the wound occurring were gathered from the DisGeNET database21, while all the 26 protein-coding genes that are participated in wound healing were gathered from the regeneration gene database [56] **(**Table 1**)**. Analyzing all these 98 genes via the DAVID database [57], resulted in identifying 15 influential genes possessing significant roles in wound generating and healing (Table S1). The GeneMANIA database reveals that six genes collaborate in angiogenesis and proliferation processes, including AKT1, HIF1A, FGF2, IGF1, TGFB1, and PIK3CA (Fig. 2). Validating obtained results via the Panther database [41], reveals four genes possessing significant roles in the angiogenesis process, including AKT1, HIF1A, FGF2, and PIK3CA, while the Enrichr database verified the obtained results **(Table S2)**.

**Table 1:** The list of 72 genes participated in the wound occurring processes gathered from DisGeNET database and 26 genes participated in the wound healing processes gathered from regeneration gene database

	Genes participated in the wound occurring processes gathered from DisGeNET database		Genes participated in the wound healing processes gathered from regeneration gene database	
No.	Gene Name	Entrez ID	Gene Name	Entrez ID
1	MMP9	4318	LTBP4	8425
2	VEGFA	7422	CXCR3	2833
3	NTS	4922	CXCR4	7852
4	KDR	3791	CRH	1392
5	GABPA	2551	LAMA5	3911
6	PTPN1	5770	PRKD1	5587
7	TGFB1	7040	PRRX1	5396
8	PIK3CA	5290	PTGDS	5730
9	PIK3CG	5294	CXCL12	6387
10	NFE2L2	4780	FERMT2	10,979
11	PIK3CD	5293	ASPRV1	151,516
12	PIK3CB	5291	IGF1	3479
13	MFGE8	4240	HOXB13	10,481
14	MMP8	4317	SKIV2L2	23,517
15	FOXO1	2308	GFPT1	2673
16	AGER	177	PRDX1	5052
17	KEAP1	9817	F13A1	2162
18	VEGFC	7424	FGF2	2247
19	CXCR4	7852	IGF2	3481
20	MIR146A	406,938	IGFBP5	3488
21	PGF	5228	LAMA3	3909
22	ANGPTL4	51,129	IL22	50,616
23	PAEP	5047	TGFB1	7040
24	SCG2	7857	VTN	7448
25	SETDB2	83,852	FCGBP	8857
26	MSC	9242	ADIPOQ	9370
27	SLC5A2	6524		
28	TNF	7124		
29	EGLN1	54,583		
30	CXCL12	6387		
31	SERPINB3	6317		
32	MOK	5891		
33	LGALS7B	653,499		
34	SP1	6667		
35	ZEB1	6935		
36	DEFB103B	55,894		
37	TET2	54,790		
38	PPARG	5468		
39	MAPK3	5595		
40	CDC25C	995		
41	MIR335	442,904		
42	FN1	2335		
43	CD93	22,918		
44	FGF7	2252		
45	ESR2	2100		
46	AKT1	207		
47	EGF	1950		
48	DSG3	1830		
49	DMBT1	1755		
50	DEFB4A	1673		
51	GADD45A	1647		
52	CCN2	1490		
53	NLRP3	114,548		
54	GPNMB	10,457		
55	MIR5591	100,847,065		
56	SIRT3	23,410		
57	SIRT1	23,411		
58	MMP1	4312		
59	DEFB103A	414,325		
60	MIR296	407,022		
61	MIR29A	407,021		
62	MIR23A	407,010		
63	MIR210	406,992		
64	MIR21	406,991		
65	MIR200B	406,984		
66	LGALS7	3963		
67	IGF1	3479		
68	HIF1A	3091		
69	GZMB	3002		
70	IL37	27,178		
71	GJA1	2697		
72	DEFB4B	100,289,462		

Using the Enrichr database, it could be possible to identify miRNAs that target the aforementioned genes (**Table S3 and S4**). Furthermore, since miRTarBase is an empirically confirmed microRNA-target interactions database [58], it has about 360.000 miRNA-target interactions (MTIs). The seed region of each miRNA is also used to determine the biological targets of miRNAs using TargetScan. Three selected miRNAs were found by checking them against the miRWalk database (Table 2). According to the mirPath v.3 database, hsa-mir-199a-3p and hsa-mir-422a have the potential to interfere with the hippo signaling pathway [59]. This route is responsible for controlling organ development, tissue regeneration, and stem cell self-renewal. YAP and TAZ, two downstream transcription coactivators with PDZ-binding motifs, govern the Hippo pathway’s essential gene regulation and biological functions [60].

**Table 2:** Three miRNAs identified via Enrichr database and verified by miRWalk database to target specified genes

miRNA	Structure	Targeted genes
hsa-mir-199a-3p	(5’-UAACCAAUGUGCAGACUACUGUGCACUAACCAAUGUGCAGACUACUGU-3’)	AKT1 FGF2
hsa-mir-422a	(5’-GCCUUCUGACCCUAAGUCCAGUGUCAGCCUUCUGACCCUAAGUCCAGU-3’)	PIK3CA AKT1
hsa-mir-4757-3p	(5’-GCGAAGCCUCUGUGACGUCAUGACAGGCGAAGCCUCUGUGACGUCAUG-3’)	AKT1 HIF1A

The antisense RNA molecule is a distinct form of DNA transcript that consists of 19–23 nucleotides and complements miRNA. Antisense RNAs regulate gene expression at numerous levels, including replication, transcription, and translation [61]. As a result, antisense miRNAs were created with the use of the microRNA sponge generator and tester (miRNAsong) program [44]. Furthermore, based on DAVID database findings, transcription factors (TFs) targeted mir-199a-3p, mir-4757-3p, and mir-422a, which play a critical role in wound healing, according to the transmiR database (Figure S1). TFs directly interpret the genome, conducting the initial step of DNA sequence decoding [62]. TFs control turning genes on and off to ensure that they are expressed in the appropriate cell at the correct time and in the proper quantity throughout the cell’s and organism’s life [62]. Based on the findings, three miRNA antisenses, hsa-mir-422a, hsa-mir-199a-3p, and hsa-mir-4757-3p, have been identified as targeting PIK3CA, FGF2, and HIF1A, respectively, while all of them target AKT1.

Wound healing is regulated by a huge proportion of cytokines and biochemicals. As a result, diabetic foot ulcer regeneration necessitates the coordinated activities of a series of diverse cell types [63]. Wounds of multiple kinds, including diabetic foot, chronic venous, and pressure ulcers, all respond favorably to the healing actions of a variety of growth factors [63]. FGFs are involved in a wide variety of developmental abnormalities, including neurodevelopment, keratinocyte organization, angiogenesis, wound healing, and any functional aberration that leads to a diversity of developmental illnesses. Angiogenesis is promoted by FGFs, which are more potent angiogenesis factors than PDGF and VEGF. As a result, granulation tissue may form as a result of angiogenesis as well as fibroblast proliferation [64].

When the expression of miRNAs linked with a given signaling pathway is reduced, the expression of signaling proteins participating in that system increases noticeably. The subsequent effects include increased cell proliferation and migration, as well as a lowered rate of apoptosis. In diabetes mellitus, reduced cell proliferation and migration, as well as accelerated death, are correlated with increased levels of miRNA proteins that are implicated in the PI3K/mTOR signaling pathway. Targeting miRNAs, which may target genes implicated in these pathways, is likely to promote proliferation and migration [65]. Moreover, Jing et al. [66] discovered that diabetic mice with low AKT/HIF-1 signaling activity had a substantial reduction in wound healing and angiogenesis.

**Fig. 3:**
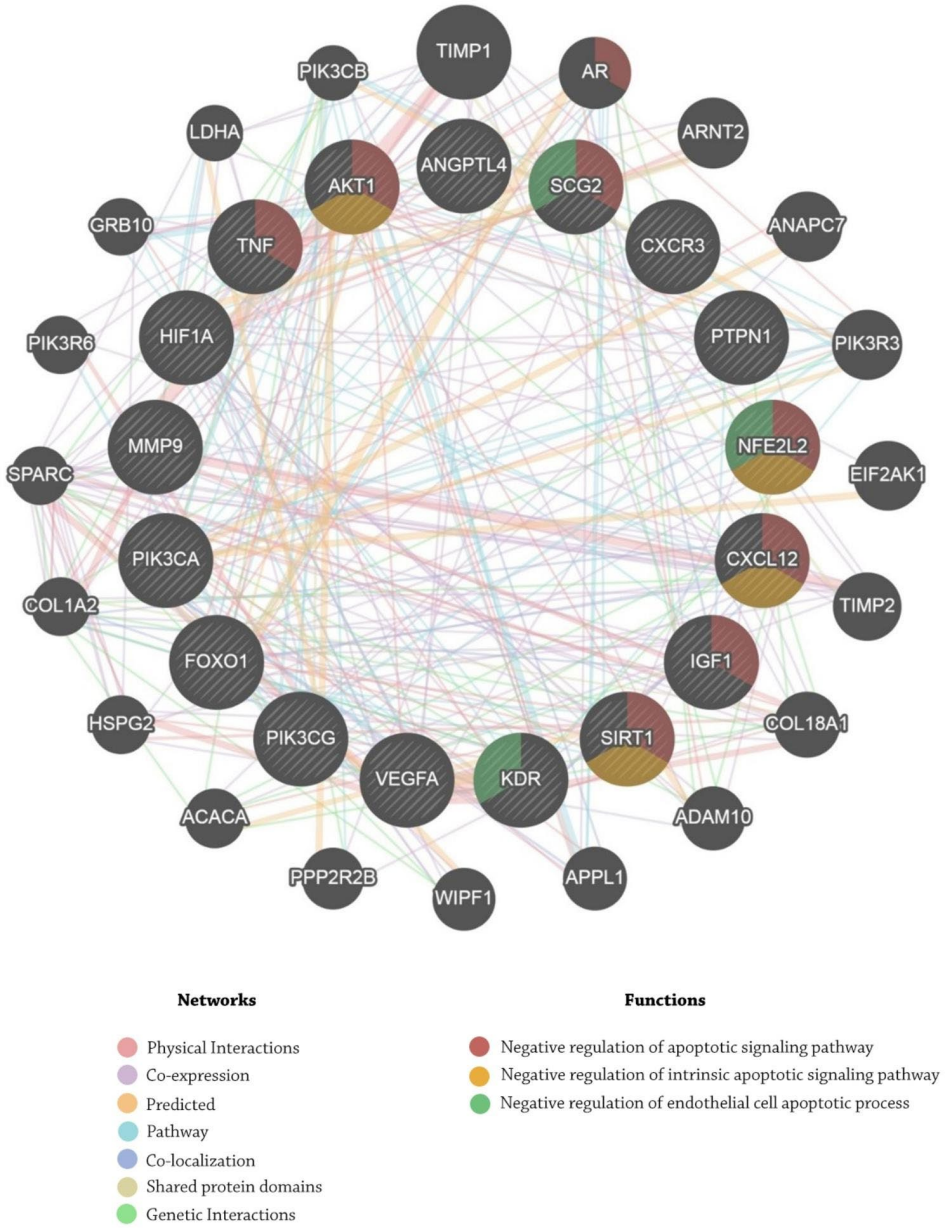
Gene ontology analysis through the GeneMANIA database resulted in demonstrating the five most influential genes, including AKT1, SCG2, NFE2L2, CXCL12, SIRT1, that are highly collaborative in negative regulation of apoptotic processes, including negative regulation of apoptotic signaling pathway, negative regulation of intrinsic apoptotic signaling pathway, negative regulation of endothelial cell apoptotic process

In terms of successful siRNA design, the DAVID database was used to identify 17 critical genes in the negative regulation of the apoptotic process (**Table S5**). The GeneMANIA database’s GO analysis revealed the five most influential genes, including AKT1, SCG2, NFE2L2, CXCL12, and SIRT1, that are highly collaborative in the negative regulation of apoptotic processes, including negative regulation of apoptotic signaling pathway, negative regulation of intrinsic apoptotic signaling pathway, and negative regulation of endothelial cell apoptotic process (Fig. 3). The Enrichr database was utilized to confirm the reported findings, which resulted in two genes being blocked by siRNAs. The Enrichr database confirmed that the NFE2L2 and SIRT1 genes play an essential part in the negative regulation of the apoptotic process; hence, these genes are the best candidates for siRNA inhibition. As a result, siRNAs were developed in the Eurofins Genomics database using the siMAX siRNA design tool (**Tables S6, S7**, and **S8**) [67]. Furthermore, nucleotide BLAST confirmed three siRNAs that had the least level of off-targeting (**Table S9**).

Figure 3.

Coagulation, inflammation, tissue formation or proliferating, and tissue remodeling or scar formation are the four permanent and temporally overlapping stages of wound healing [68]. During the apoptotic phase, neutrophils are absorbed by macrophages to prevent further inflammation [69]. Apoptosis in immune cells may be the key to ending inflammation and kicking off healing since macrophages are the major phagocytes that help resolve inflammation and promote tissue regeneration [70]. Diabetes reduces the number and activity of macrophages, which in turn reduces the development of lymphatic vessels and hence slows the healing process. NFE2L2 and SIRT1 are two key genes that regulate apoptosis and might be targeted to improve tissue regeneration [71].

### Molecular dynamics (MD) simulation

Based on input data provided in Table 3, MD simulation gives the parameters of length, mass, net charge, and hydrophobicity of each RNAis. The pH and temperature at the diabetic wound area were considered as 6.95 and 30.91 °C, respectively [54]. The key parameters obtained from the software show appropriately the details of the structures of siRNA, miRNA and nanocarriers. Table 3 lists the full names and specifications of each structure.

**Table 3:** Molecular dynamics simulation inputs and structures

Material	No.	Structure
miRNA Antisense	1	422a (5’-GCCUUCUGACCCUAAGUCCAGUGUCAGCCUUCUGACCCUAAGUCCAGU-3’)
	2	4757-3p (5’-GCGAAGCCUCUGUGACGUCAUGACAGGCGAAGCCUCUGUGACGUCAUG-3’)
	3	199a-3p (5’-UAACCAAUGUGCAGACUACUGUGCACUAACCAAUGUGCAGACUACUGU-3’)
SiRNA	4	CCAGUUGACAGUGAACUCA (for NFE2L2 gene)
	5	GUUGACCUCCUCAUUGUUA (for SIRT1 gene)
	6	GUAAGACCAGUAGCACUAA (for SIRT1 gene)
Nanocarriers	a	Polyethylenimines (PEIs)
	b	poly(lactide-co-glycolide) (PLGA)
	c	Chitosan

**Table S10** also shows the basic parameters for all six structures of siRNA and miRNA antagomirs, such as the mass and net charge of the molecules, so that they can be used to interpret the output analyses obtained from the simulation of MD. Moreover, Table 4 presents the average amount of Van der Waals and electrostatic energies, total energy, Gyration radius, and solvent-accessible surface area (SASA) for each nanocarrier, miRNAs, and siRNAs.

**Table 4:** The results of interactions among carriers and miRNA antisenses and SiRNAs

Parameter	Carrier	miRNA Antisense			SiRNA		
		1	2	3	4	5	6
VDW Energy (KJ/mol)	PLGA	-1201.15	-952.161	-1114.46	-811.154	-703.456	-759.564
	PEI	-801.744	-603.567	-704.415	-484.188	-440.485	-451.745
	Chitosan	-425.654	-302.485	-397.561	-156.546	-108.584	-146.474
Electrostatics Energy (KJ/mol)	PLGA	-1.465	-1.158	-1.164	-0.985	-1.007	-0.752
	PEI	-7.465	-3.594	-6.654	-1.176	-2.841	-1.054
	Chitosan	170.251	128.214	164.214	77.547	83.147	54.173
Total Energy (KJ/mol)	PLGA	-1202.62	-953.319	-1115.62	-812.139	-704.463	-760.316
	PEI	-809.209	-607.161	-711.069	-485.364	-443.326	-452.799
	Chitosan	-255.403	-174.271	-233.347	-78.999	-25.437	-92.301
Gyrate radius (Initial-Final) (nm)	PLGA	2.154	2.048	2.117	1.945	1.744	1.872
	PEI	1.844	1.485	1.737	0.907	0.732	0.841
	Chitosan	0.471	0.257	0.343	0.097	0.047	0.14
SASA (nm^2)	PLGA	408.416	397.465	404.489	370.214	364.45	367.312
	PEI	350.215	339.764	347.526	307.774	298.117	301.731
	Chitosan	284.247	269.564	274.337	209.15	204.563	218.416

The van der Waals energies are related to the interactions among atoms, which vary according to the size and mass of each atom. This means that in this analysis, the higher the mass and number of atoms of a molecule, the more van der Waals energy it can have. The calculation of these energies in MD follows the Lennard-Jones equation [72]. Table 4 clearly shows that miRNAs exhibit stronger van der Waals interactions than siRNAs. In general, according to the mass data shown in **Table S10**, the higher the mass of the molecules, the more van der Waals energy they could have, depending on the type of nanocarrier.

Electrostatic energies are also a function of the net charge of the atoms of each molecule, meaning that molecules that have higher and unlike charges have more electrostatic energy. Molecules that have like charges also possess positive electrostatic energy. In MD, electrostatic energy also follows Coulomb’s law [73]. Therefore, a comparison could be available for these simulations according to the net charge of siRNA and miRNA molecules reported in Table 4. From this comparison, it could be concluded that the net charge of miRNAs is higher than that of siRNAs, and the ratio of their electrostatic energy to the molecular charge of these structures has changed.

Based on the electrostatic energy, Poly (lactic-co-glycolic acid) (PLGA), Polyethylenimine (PEI), and Chitosan nanocarriers were able to have better electrostatic absorption for structures, respectively. The electrostatic adsorption energy is positive for the Chitosan nanocarrier, and this is an unsuitable parameter for the adsorption between the Chitosan nanocarrier and the RNA structures. The total energy is also calculated from the sum of van der Waals and electrostatic energies and is a complete indicator for calculating the amount of adsorption among RNA structures and nanocarriers. Based on the total energy, the PLGA nanocarriers had better adsorption than PEI and Chitosan. Also, miRNAs 1 and 3 had the best adsorption; hence, the highest adsorption energy was dedicated to the adsorption among PLGA and miRNA No. 1, as well as PLGA and miRNA No. 3.

These results are also validated through the analysis of Gyration radius and SASA. The average Gyration radius for the simulations also shows a better aggregation of RNA structures and nanocarriers. The lower value results in more significant aggregation and adsorption. This analysis positively correlates to the results obtained from the total energy analyses.

**Fig. 4:**
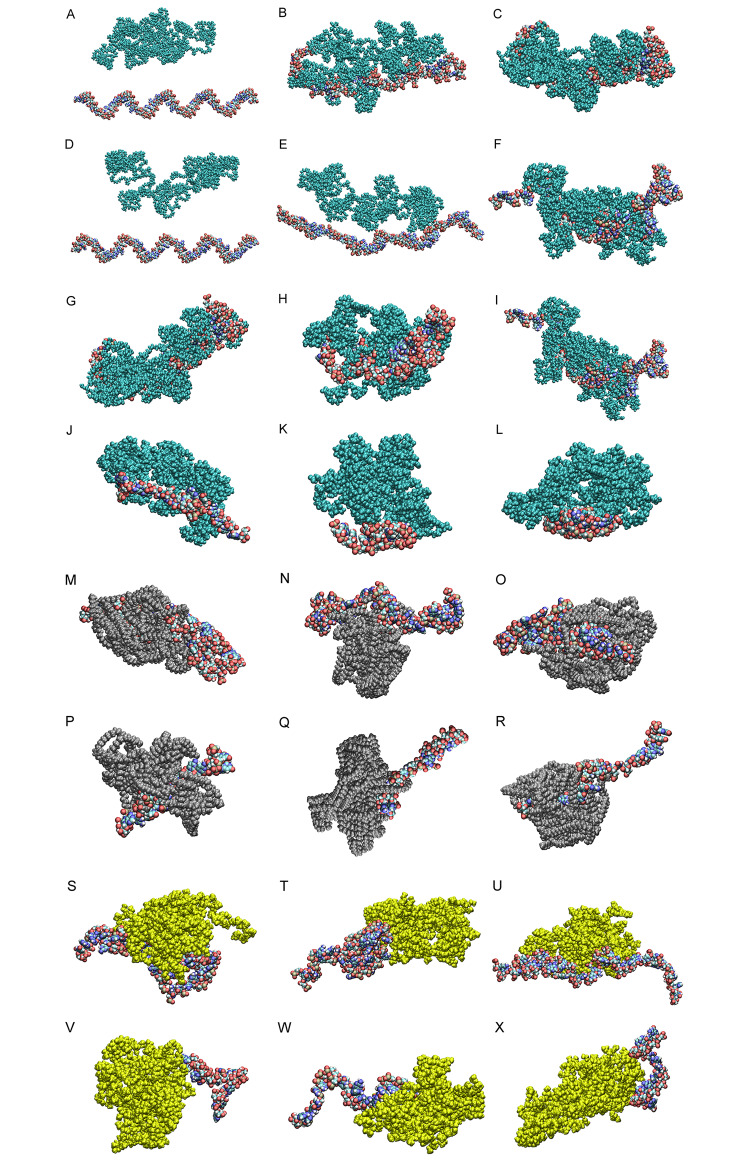
The snapshots of MD simulations among (**A-C**) miRNA antisense 1/PLGA carrier, and (**D-F**) miRNA antisense 3/PLGA carrier in the (**A** and **D**) input, (**B** and **E**) middle, and (**C** and **F**) output modes of the simulation. Figures (**G-I**) show the output interaction modes of (**G**) miRNA antisense 1, (**H**) miRNA antisense 2, (**I**) miRNA antisense 3, (**J**) SiRNA 1, (**K**) SiRNA 2, (**L**) SiRNA 3, with PLGA carrier. In this figures, dark green, red, and light green molecules represent PLGA polymer, Oxygen atoms, and Carbon atoms, while white, blue, and light brown represent Hydrogen, Nitrogen, and Phosphorus atoms, respectively. Figures (**M-R**) show the output interaction modes of (**M**) miRNA antisense 1, (**N**) miRNA antisense 2, (**O**) miRNA antisense 3, (**P**) SiRNA 1, (**Q**) SiRNA 2, (**R**) SiRNA 3, with PEI carrier, while Figures (**S-X**) show the output interaction modes of (**S**) miRNA antisense 1, (**T**) miRNA antisense 2, (**U**) miRNA antisense 3, (**V**) SiRNA 1, (**W**) SiRNA 2, (**X**) SiRNA 3, with Chitosan carrier. In this Figures, silver, yellow, red, and light green molecules represent PEI polymer, Chitosan polymer, Oxygen, and Carbon atoms, while white, blue, and light brown represent Hydrogen, Nitrogen, and Phosphorus atoms, respectively

SASA analysis also shows the surface in contact with the solvent for the structures. Changes in this analysis are also done according to the size of the RNA structures and nanocarriers and the degree of hydrophilicity and hydrophobicity of the molecules. Figure 4 A-F show different stages of simulation for nanocarriers and RNAi structures. It is possible to compare the amount of adsorption in various simulation phases, including input, middle, and output. The analyses were pursued through output phases regarding the superior influence of output mode over input and middle phases in MD simulations. Figure 4G-L demonstrate the output interaction modes of PLGA nanocarriers and RNAis.

**Fig. 5:**
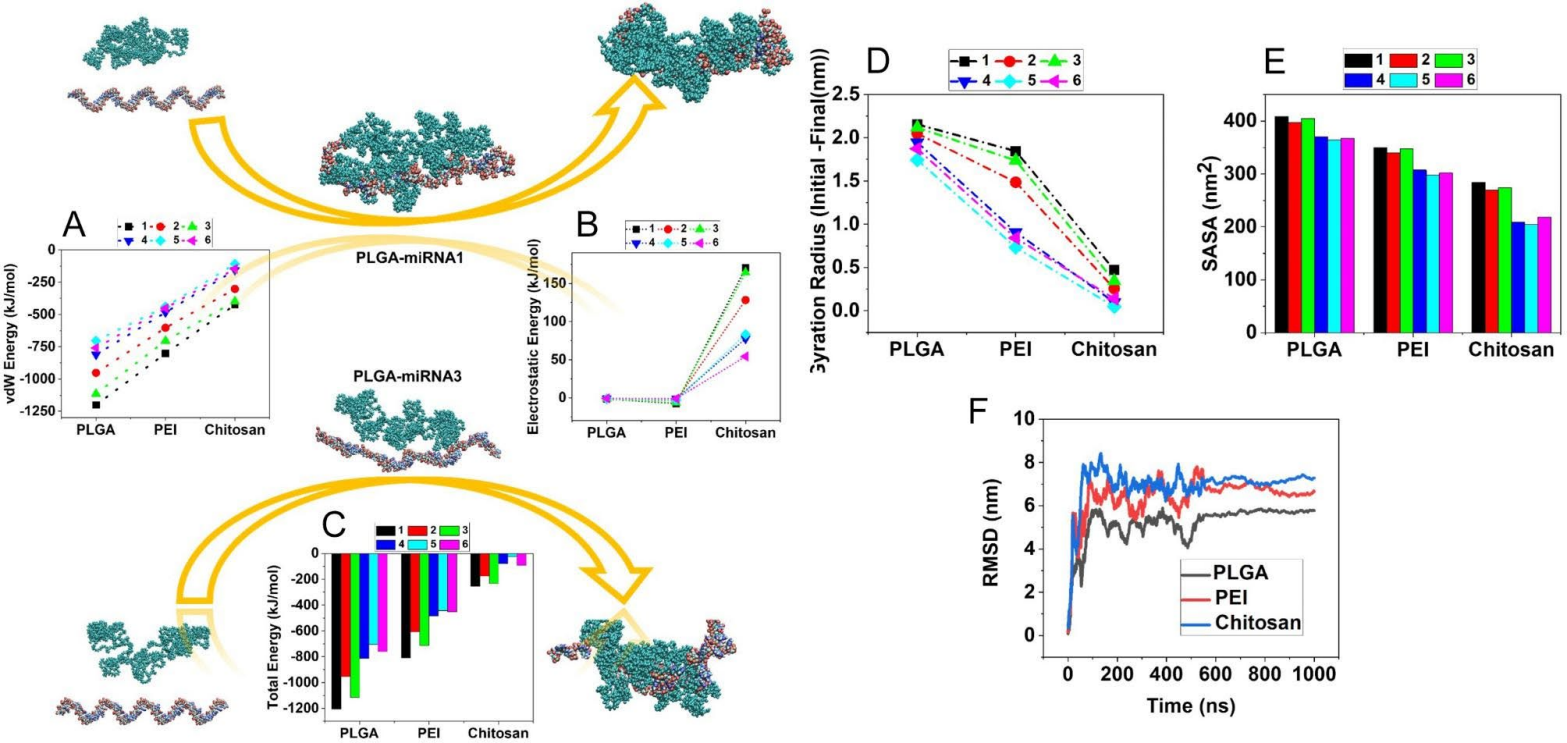
Energy analysis in bioresponsive polymeric nanocarriers and RNAis interaction. (**A**) van der Waals interaction energy of PLGA, PEI, and Chitosan with miRNA antisenses and siRNAs, (**B**) Electrostatic interaction energy of PLGA, PEI, and Chitosan with miRNA antisenses and siRNAs, (**C**) Total interaction energy of PLGA, PEI, and Chitosan with miRNA antisenses and siRNAs, (**D**) Different between initial and final Gyration radius of loaded miRNA antisenses and siRNAs, (**E**) Average SASA of loaded miRNA antisenses and siRNAs, (**F**) RMSD of loaded miRNA antisenses (1) during the MD simulation

The output interaction modes of PEI and Chitosan nanocarriers with RNAi structures are available in Fig. 4M-X. The integration of RNAis and polymeric nanocarriers in these figures investigates that PEI is more integrated with miRNA antagomirs and siRNAs than Chitosan. The comparison of Figs. 4G-L and 5 led to discuss the stability, van der Waals, electrostatic, and total energies in MD simulations. Figure 5 A demonstrates the van der Waals interaction energy of PLGA, PEI, and Chitosan with miRNA antisenses and siRNAs, introducing the integration of PLGA with miRNA antagomir 1 as the optimum delivery system in terms of van der Waals interaction energy. Furthermore, Fig. 5B indicates electrostatic energy, in which the PLGA and PEI are in a similar negative range, while Chitosan is in the positive area, with a severe discrepancy. Finally, the total energy is shown in Fig. 5C, which summarizes all the given analyses in the energy area. Based on this chart, the integration of PLGA is more desirable than other polymeric nanocarriers, while delivering the miRNA antagomirs is more stable than siRNAs to the diabetic ulcer areas.

To confirm the obtained results, analyses of the Gyration radius and SASA are performed and presented in Fig. 5D and E, respectively. The Gyration radius of the PLGA/ miRNA antagomir 1 combination (2.154 nm) is higher than others, while its higher SASA amount (408.416 nm^2) confirms that. Therefore, it would be better to investigate its root-mean-square deviation (RMSD) along the MD simulation (Fig. 5F**)**. RMSD is a prominent stability measure in MD, representing the particle position deviation concerning their reference position [74]. The RMSD results positively correlate with Gyration radius analysis regarding the hydrophilicity performance. Finally, **Table S11** summarizes the results according to the MD simulation output analyzes of RNAis and nanocarriers, considering the priority sorted from the highest adsorption to the lowest one.

## Conclusions

A systems biology approach was employed in the current work to investigate how some RNAis promote diabetic ulcer regeneration by improving angiogenesis. Moreover, to discover the stable delivery of proposed miRNAs and siRNAs into the wound area, their aggregation with three bioresponsive polymeric nanocarriers (i.e., PLGA, PEI, and Chitosan) were studied through MD simulation. Multiple self-assembly parameters and stable integration were examined, including van der Waals energy, electrostatic energy, total energy, Gyration radius, SASA, and RMSD. Results demonstrate that PLGA, PEI, and Chitosan possess the lowest energy, leading to more stability and safe delivery, respectively. Also, results show that miRNAs prioritize utilize as the therapeutic agents compared with siRNAs. It is proposed that as poor angiogenesis during diabetic wound healing is linked to either a lack of pro-angiogenic factors or an increase in anti-angiogenic factors, improving promoters and decreasing suppressors of the angiogenesis process could accelerate wound healing in diabetic ulcers. The results from the systems biology and MD studies led us to choose utilizing PLGA nanocarrier to deliver the hsa-mir-422a antagomir as the most stable condition to recommend for further development and improvement of angiogenesis applications.

## Electronic supplementary material

Below is the link to the electronic supplementary material.

Supplementary Material 1

## Data Availability

The datasets used and/or analyzed during the current study are available from the corresponding author (Nima Beheshtizadeh) on reasonable request.

## Abbreviations

DAVID: Database for annotation, visualization and integrated discovery
FGF: Fibroblast growth factors
GO: Gene ontology
HIF-1: Hypoxia-inducible factor
MMP9: Matrix metalloproteinase
PLGA: Poly(lactic-co-glycolic acid)
PHD-2: Prolyl-hydroxylase domain 2
Panther: Protein analysis through evolutionary relationships
RNAi: RNA interference
RNA: Ribonucleic acid
siRNA: Small interfering RNA
SASA: Solvent-accessible surface area
TFs: Transcription factors
TAZ: Transcriptional coactivator with PDZ-binding motif
TGF- β: Transforming growth factor β
VEGF: Vascular endothelial growth factor
YAP: Yes-associated protein
miRNA: microRNA
