# Supplementary material for: In-silico engineering of RNA nanoplatforms to promote the diabetic wound healing
Source: BMC Chem. 2023 Jun 8;17(1):52. doi: 10.1186/s13065-023-00969-4 (PMC10251717; doi:10.1186/s13065-023-00969-4)
Supplement: Supplementary file 1 — Supplementary Material 1 [file 13065_2023_969_MOESM1_ESM.docx]

In-silico engineering of RNA nanoplatforms to promote the diabetic wound healing

Nima Beheshtizadeh^1,5*^, Alireza Salimi^2,5^, Mahsa Golmohammadi^3^, Javad Mohajer Ansari^4,5^, Mahmoud Azami^1,5**^

^1^ Department of Tissue Engineering, School of Advanced Technologies in Medicine, Tehran University of Medical Sciences, Tehran, Iran

^2^ Department of Advanced Technologies, School of Medicine, North Khorasan University of Medical Science, Bojnurd, Iran

^3^ Department of Polymer Engineering and Color Technology, Amirkabir University of Technology, Tehran, Iran

^4^ Department of Anatomy, School of Medicine, Hormozgan University of Medical Sciences, Jomhuri Eslami Blvd, Bandar Abbas, 7919915519, Iran

^5^ Regenerative Medicine group (REMED), Universal Scientific Education and Research Network (USERN), Tehran, Iran

Corresponding authors:

*Nima Beheshtizadeh (n-beheshtizadeh@razi.tums.ac.ir)

Address: No. 88, Italia St, Qods Ave, Keshavarz Blvd, Tehran, 14177-55469, Iran.

**Mahmoud Azami (m-azami@tums.ac.ir)

Address: No. 88, Italia St, Qods Ave, Keshavarz Blvd, Tehran, 14177-55469, Iran.


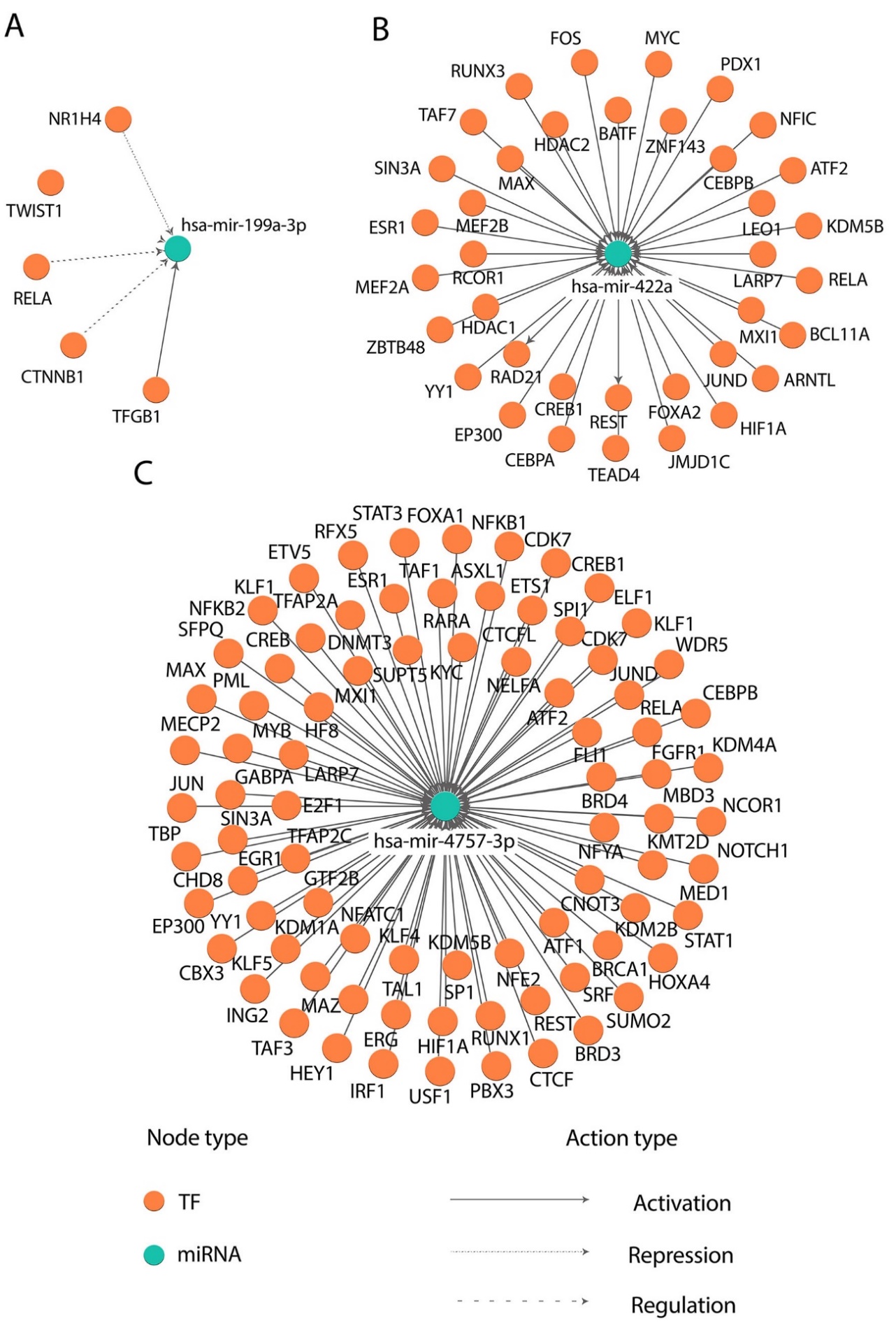


­**Figure S1.** (**A**) TFs which have interaction with hsa-mir 199a-3p, (**B**) TFs which have interaction with hsa-mir-422a, (**C**) TFs which have interaction with hsa-mir-4757-3p.

**Table S1.** The list of protein coding genes resulted via DAVID analyses

| No. | Gene Name | Entrez ID |
| --- | --- | --- |
| 1 | AKT1 | 207 |
| 2 | AGER | 177 |
| 3 | F13A1 | 2162 |
| 4 | FGF2 | 2247 |
| 5 | FN1 | 2335 |
| 6 | GJA1 | 2697 |
| 7 | HIF1A | 3091 |
| 8 | IGF1 | 3479 |
| 9 | MAPK3 | 5595 |
| 10 | NFE2L2 | 4780 |
| 11 | PIK3CA | 5290 |
| 12 | PIK3CB | 5291 |
| 13 | PIK3CG | 5294 |
| 14 | TGFB1 | 7040 |
| 15 | VTN | 7448 |

**Table S2.** Enrichr database verified the obtained results via determining the influential processes in angiogenesis based on gene ontology

| **No.** | **Function** | **P-value** | **Q-value** | **Active genes** |
| --- | --- | --- | --- | --- |
| 1 | Positive regulation of blood vessel endothelial cell migration (GO:0043536) | 4.362992e-10 | 2.686564e-07 | TGFB1, AKT1, FGF2, HIF1A |
| 2 | Regulation of blood vessel endothelial cell migration (GO:0043535) | 7.643141e-10 | 2.686564e-07 | TGFB1, AKT1, FGF2, HIF1A |
| 3 | Positive regulation of phosphorylation (GO:0042327) | 1.849083e-09 | 4.333019e-07 | TGFB1, AKT1, IGF1, FGF2, HIF1A |
| 4 | Positive regulation of endothelial cell migration (GO:0010595) | 4.747293e-09 | 8.343368e-07 | TGFB1, AKT1, FGF2, HIF1A |
| 5 | Positive regulation ofmacromolecule metabolic process (GO:0010604) | 1.501545e-08 | 2.111172e-06 | TGFB1, AKT1, IGF1, FGF2, HIF1A |
| 6 | Positive regulation of epithelial cell proliferation (GO:0050679) | 2.023593e-08 | 2.370976e-06 | AKT1, IGF1, FGF2, HIF1A |
| 7 | Protein kinase B signalling (GO:0043491) | 3.028853e-08 | 3.041834e-06 | TGFB1, AKT1, IGF1 |
| 8 | Positive regulation of gene expression (GO:0010628) | 4.684198e-08 | 3.658879e-06 | TGFB1, AKT1, IGF1, FGF2, HIF1A |
| 9 | Cellular response to cytokine stimulus (GO:0071345) | 4.684198e-08 | 3.658879e-06 | TGFB1, PIK3CA, AKT1, FGF2, HIF1A |
| 10 | Positive regulation of nucleic acid-templated transcription (GO:1903508) | 6.273143e-08 | 4.410019e-06 | TGFB1, AKT1, IGF1, FGF2, HIF1A |

**Table S3.** Identifying miRNAs, through Enrichr database and miRTarBase tool

| **No.** | **miRNAs** | **P-value** | **Q-value** | **Targeted genes** |
| --- | --- | --- | --- | --- |
| 1 | hsa-miR-422a | 2.118537e-07 | 0.000085 | TGFB1, PIK3CA, AKT1 |
| 2 | hsa-miR-199a-3p | 3.562375e-06 | 0.000662 | AKT1, IGF1, FGF2 |
| 3 | hsa-miR-4757-3p | 0.002398 | 0.05670 | AKT1 |
| 4 | mmu-miR-18a-5p | 4.943298e-06 | 0.000662 | IGF1, HIF1A |
| 5 | mmu-miR-101a-3p | 2.623728e-05 | 0.002637 | AKT1, HIF1A |
| 6 | hsa-miR-625-3p | 5.530158e-05 | 0.003857 | AKT1, HIF1A |
| 7 | hsa-miR-155-5p | 5.756402e-05 | 0.003857 | PIK3CA, AKT1, FGF2, HIF1A |
| 8 | hsa-miR-18b-5p | 4.927044e-04 | 0.025949 | IGF1, HIF1A |
| 9 | hsa-miR-138-5p | 5.717949e-04 | 0.025949 | AKT1, HIF1A |
| 10 | hsa-miR-302a-5p | 5.809398e-04 | 0.025949 | IGF1, FGF2 |

**Table S4.** Identifying miRNAs, through Enrichr database and TargetScan tool

| No. | miRNAs | P-value | Q-value | Targeted genes |
| --- | --- | --- | --- | --- |
| 1 | hsa-miR-564 | 0.0036501 | 0.215096 | TGFB1, AKT1, FGF2 |
| 2 | mmu-miR-151-3p | 0.0071533 | 0.215096 | IGF1, FGF2, HIF1A |
| 3 | hsa-miR-4473 | 0.0072883 | 0.215096 | AKT1, IGF1, FGF2 |
| 4 | mmu-miR-5108 | 0.0087716 | 0.215096 | PIK3CA, IGF1, FGF2 |
| 5 | mmu-miR-5116 | 0.009064 | 0.215096 | IGF1, FGF2, HIF1A |
| 6 | mmu-miR-155 | 0.0092046 | 0.215096 | IGF1, FGF2, HIF1A |
| 7 | mmu-miR-18b | 0.0100431 | 0.215096 | IGF1, FGF2, HIF1A |
| 8 | mmu-miR-18a | 0.0100431 | 0.215096 | IGF1, FGF2, HIF1A |
| 9 | mmu-miR-378b | 0.0102263 | 0.215096 | PIK3CA, AKT1, IGF1 |
| 10 | mmu-miR-3092 | 0.0395648 | 0.277829 | AKT1, FGF2 |

**Table S5.** Essential genes in negative regulation of apoptotic process, identified through DAVID database

| No. | Gene Name | Entrez ID |
| --- | --- | --- |
| 1 | NRF2 | 4780 |
| 2 | CXCL12 | 6387 |
| 3 | CXCR3 | 2833 |
| 4 | ANGPTL4 | 51129 |
| 5 | FOXO1 | 2308 |
| 6 | HIF1A | 3091 |
| 7 | KDR | 3791 |
| 8 | PIK3CG | 5294 |
| 9 | PIK3CA | 5290 |
| 10 | PTPN1 | 5770 |
| 11 | SCG2 | 7857 |
| 12 | SIRT1 | 23411 |
| 13 | AKT | 207 |
| 14 | TNF | 7124 |
| 15 | VEGFA | 7422 |
| 16 | MMP9 | 4318 |
| 17 | IGF1 | 3479 |

**Table S6.** Designing siRNAs for targeting NFE2L2 gene

| **No.** | **siRNA Sequence (Sense)** | **mRNA Motif** | **start c** | **stop c** | **GC** | **Dsir-Score** | **Ui-Tei Score** | **Reynolds Score** |
| --- | --- | --- | --- | --- | --- | --- | --- | --- |
| 1 | GCACAGCAGAAUUCAAUGA | AAGCACAGCAGAAUUCAAUGAUU | 1001 | 818 | 42.1 | 92.2 | Ia | 7 |
| 2 | CCAGUUGACAGUGAACUCA | AACCAGUUGACAGUGAACUCAUU | 789 | 1030 | 47.4 | 84.6 | Ib | 5 |

**Table S7.** Designing siRNAs for targeting SIRT1 gene

| **No.** | **siRNA Sequence (Sense)** | **mRNA Motif** | **start c** | **stop c** | **GC** | **Dsir-Score** | **Ui-Tei Score** | **Reynolds Score** |
| --- | --- | --- | --- | --- | --- | --- | --- | --- |
| 1 | GUUGACCUCCUCAUUGUUA | AAGUUGACCUCCUCAUUGUUAUU | 409 | 951 | 42.1 | 88.4 | Ia | 6 |
| 2 | GUAAGACCAGUAGCACUAA | AAGUAAGACCAGUAGCACUAAUU | 445 | 915 | 42.1 | 91.4 | Ib | 6 |
| 3 | GAACUUCACCACCAGAUUC | AAGAACUUCACCACCAGAUUCUU | 740 | 620 | 47.4 | 76.2 | II | 5 |
| 4 | GAUGAAGUUGACCUCCUCA | AAGAUGAAGUUGACCUCCUCAUU | 403 | 957 | 47.4 | 89.8 | II | 2 |
| 5 | GGAUAAUUCAGUGUCAUGG | AAGGAUAAUUCAGUGUCAUGGUU | 185 | 1175 | 42.1 | 73.4 | II | 1 |

**Table S8.** Selected siRNAs for targeting NFE2L2and SIRT1 genes

| No. | siRNA Sequence (Sense) | mRNA Motif | gene |
| --- | --- | --- | --- |
| 1 | CCAGUUGACAGUGAACUCA | AACCAGUUGACAGUGAACUCAUU | NFE2L2 |
| 2 | GUUGACCUCCUCAUUGUUA | AAGUUGACCUCCUCAUUGUUAUU | SIRT1 |
| 3 | GUAAGACCAGUAGCACUAA | AAGUAAGACCAGUAGCACUAAUU | SIRT1 |

**Table S9.** Three siRNAs designed by siMAX siRNA Design tool to target specified genes

| **siRNA** | **Targeted genes** |
| --- | --- |
| CCAGUUGACAGUGAACUCA | NFE2L2 |
| GUUGACCUCCUCAUUGUUA | SIRT1 |
| GUAAGACCAGUAGCACUAA | SIRT1 |

**Table S10.** Obtained parameters of molecular dynamics simulations

| **Parameter** | **miRNA Antisense** | | | **SiRNA** | | |
| --- | --- | --- | --- | --- | --- | --- |
| RNA interaction number | 1 | 2 | 3 | 4 | 5 | 6 |
| Length | 49 | 49 | 50 | 30 | 19 | 30 |
| Mass | 4955.12 | 4529.408 | 4812.4 | 3263.88 | 2124.859 | 3106.019 |
| Net charge | -14 | -10 | -12 | -6 | -8 | -3 |
| Hydrophobicity (Kcal/mol) | 64.51 | 62.03 | 64.55 | 39.08 | 38.35 | 36.78 |

**Table S11.** Priority of employing miRNA antisenses and SiRNAs in combining with nanocarriers

| **Carrier** | **miRNA Antisense** | | | | **SiRNA** | | |
| --- | --- | --- | --- | --- | --- | --- | --- |
|  | **1** | **2** | **3** | **4** | | **5** | **6** |
| PLGA | 1 | 3 | 2 | 4 | | 6 | 5 |
| PEI | 7 | 9 | 8 | 10 | | 12 | 11 |
| Chitosan | 13 | 15 | 14 | 17 | | 18 | 16 |

Available raw data for this study

Table A1. Protein class- Cell adhesion

| **Disease** | **Disease_id** | **Gene** | **Gene_id** | **UniProt** | **Gene_Full_Name** | **Protein_Class** | **N_diseases_g** | **DSI_g** | **DPI_g** | **pLI** | **Score_gda** | **EI_gda** | **N_SNPs_gda** | **First_Ref** | **Last_Ref** |
| --- | --- | --- | --- | --- | --- | --- | --- | --- | --- | --- | --- | --- | --- | --- | --- |
| Diabetic wound | C4728046 | DSG3 | 1830 | P32926 | desmoglein 3 | Cell adhesion | 78 | 0.603 | 0.654 | 1.46E-20 | 0.01 | 1 | 0 | 2018 | 2018 |

Table A2. Protein class-cell to cell junction

| **Disease** | **Disease_id** | **Gene** | **Gene_id** | **UniProt** | **Gene_Full_Name** | **Protein_Class** | **N_diseases_g** | **DSI_g** | **DPI_g** | **pLI** | **Score_gda** | **EI_gda** | **N_PMIDs** | **N_SNPs_gda** | **First_Ref** | **Last_Ref** |
| --- | --- | --- | --- | --- | --- | --- | --- | --- | --- | --- | --- | --- | --- | --- | --- | --- |
| Diabetic wound | C4728046 | DSG3 | 1830 | P32926 | desmoglein 3 | Cell adhesion | 78 | 0.603 | 0.654 | 1.46E-20 | 0.01 | 1 | 1 | 0 | 2018 | 2018 |

Table A3. Protein class- Nucleic acid binding

| **Disease** | **Disease_id** | **Gene** | **Gene_id** | **UniProt** | **Gene_Full_Name** | **Protein_Class** | **N_diseases_g** | **DSI_g** | **DPI_g** | **pLI** | **Score_gda** | **EI_gda** | **N_PMIDs** | **N_SNPs_gda** | **First_Ref** | **Last_Ref** |
| --- | --- | --- | --- | --- | --- | --- | --- | --- | --- | --- | --- | --- | --- | --- | --- | --- |
| Diabetic wound | C4728046 | SP1 | 6667 | P08047 | Sp1 transcription factor | Nucleic acid binding | 209 | 0.493 | 0.769 | 0.99997 | 0.01 | 1 | 1 | 0 | 2018 | 2018 |

Table A4. Protein class- G-protein coupled receptor

| **Disease** | **Disease_id** | **Gene** | **Gene_id** | **UniProt** | **Gene_Full_Name** | **Protein_Class** | **N_diseases_g** | **DSI_g** | **DPI_g** | **pLI** | **Score_gda** | **EI_gda** | **N_PMIDs** | **N_SNPs_gda** | **First_Ref** | **Last_Ref** |
| --- | --- | --- | --- | --- | --- | --- | --- | --- | --- | --- | --- | --- | --- | --- | --- | --- |
| Diabetic wound | C4728046 | CXCR4 | 7852 | P61073 | C-X-C motif chemokine receptor 4 | G-protein coupled receptor | 739 | 0.362 | 0.923 | 0.017804 | 0.01 | 1 | 1 | 0 | 2017 | 2017 |

Table A5. Protein class-Transporter

| **Disease** | **Disease_id** | **Gene** | **Gene_id** | **UniProt** | **Gene_**  **Full_Name** | **Protein_Class** | **N_diseases_g** | **DSI_g** | **DPI_g** | **pLI** | **Score_gda** | **EI_gda** | **N_PMIDs** | **N_SNPs_gda** | **First_Ref** | **Last_Ref** |
| --- | --- | --- | --- | --- | --- | --- | --- | --- | --- | --- | --- | --- | --- | --- | --- | --- |
| Diabetic wound | C4728046 | SLC5A2 | 6524 | P31639 | solute carrier family 5 member 2 | Transporter | 214 | 0.499 | 0.769 | 4.92E-14 | 0.01 | 1 | 1 | 0 | 2019 | 2019 |

Table A6. Protein class- Enzyme modulator

| **Disease** | **Disease_id** | **Gene** | **Gene_id** | **UniProt** | **Gene_Full**  **_Name** | **Protein_Class** | **N_diseases_g** | **DSI_g** | **DPI_g** | **pLI** | **Score_gda** | **EI_gda** | **N_PMIDs** | **N_SNPs_gda** | **First_Ref** | **Last_Ref** |
| --- | --- | --- | --- | --- | --- | --- | --- | --- | --- | --- | --- | --- | --- | --- | --- | --- |
| Diabetic wound | C4728046 | SERPINB3 | 6317 | P29508 | serpin family B member 3 | Enzyme modulator | 101 | 0.565 | 0.615 | 8.21E-16 | 0.01 | 1 | 1 | 0 | 2014 | 2014 |

Table A7. Protein class- Epigenetic regulator

| **Disease** | **Disease_id** | **Gene** | **Gene_id** | **UniProt** | **Gene_Full_Name** | **Protein_Class** | **N_diseases_g** | **DSI_g** | **DPI_g** | **pLI** | **Score_gda** | **EI_gda** | **N_PMIDs** | **N_SNPs_gda** | **First_Ref** | **Last_Ref** |
| --- | --- | --- | --- | --- | --- | --- | --- | --- | --- | --- | --- | --- | --- | --- | --- | --- |
| Diabetic wound | C4728046 | SIRT3 | 23410 | Q9NTG7 | sirtuin 3 | Epigenetic regulator | 227 | 0.488 | 0.731 | 0.000163 | 0.01 | 1 | 1 | 0 | 2019 | 2019 |
| Diabetic wound | C4728046 | SIRT1 | 23411 | Q96EB6 | sirtuin 1 | Epigenetic regulator | 675 | 0.378 | 0.885 | 0.087326 | 0.01 | 1 | 1 | 0 | 2019 | 2019 |

Table A8. Protein class- nuclear receptor

| **Disease** | **Disease_id** | **Gene** | **Gene_id** | **UniProt** | **Gene_Full_Name** | **Protein_Class** | **N_diseases_g** | **DSI_g** | **DPI_g** | **pLI** | **Score_gda** | **EI_gda** | **N_PMIDs** | **N_SNPs**  **_gda** | **First_Ref** | **Last_Ref** |
| --- | --- | --- | --- | --- | --- | --- | --- | --- | --- | --- | --- | --- | --- | --- | --- | --- |
| Diabetic wound | C4728046 | PPARG | 5468 | P37231 | peroxisome proliferator activated receptor gamma | Nuclear receptor | 877 | 0.358 | 0.885 | 0.029194 | 0.01 | 1 | 1 | 0 | 2015 | 2015 |
| Diabetic wound | C4728046 | ESR2 | 2100 | Q92731 | estrogen receptor 2 | Nuclear receptor | 528 | 0.4 | 0.923 | 4.45E-08 | 0.01 | 1 | 1 | 0 | 2019 | 2019 |

Table A9. Protein class- Transcription factor

| **Disease** | **Disease_id** | **Gene** | **Gene_id** | **UniProt** | **Gene_Full_Name** | **Protein_Class** | **N_diseases_g** | **DSI_g** | **DPI_g** | **pLI** | **Score_gda** | **EI_gda** | **N_PMIDs** | **N_SNPs_gda** | **First_Ref** | **Last_Ref** |
| --- | --- | --- | --- | --- | --- | --- | --- | --- | --- | --- | --- | --- | --- | --- | --- | --- |
| Diabetic wound | C4728046 | GABPA | 2551 | Q06546 | GA binding protein transcription factor subunit alpha | Transcription factor | 632 | 0.379 | 0.885 | 0.99812 | 0.02 | 1 | 2 | 0 | 2016 | 2018 |
| Diabetic wound | C4728046 | ZEB1 | 6935 | P37275 | zinc finger E-box binding homeobox 1 | Transcription factor | 310 | 0.442 | 0.808 | 0.96665 | 0.01 | 1 | 1 | 0 | 2019 | 2019 |
| Diabetic wound | C4728046 | HIF1A | 3091 | Q16665 | hypoxia inducible factor 1 subunit alpha | Transcription factor | 1044 | 0.327 | 0.923 | 0.9777 | 0.01 | 1 | 1 | 0 | 2017 | 2017 |

Table A10. Protein class-Receptor

| **Disease** | **Disease_id** | **Gene** | **Gene_id** | **UniProt** | **Gene_Full_Name** | **Protein_Class** | **N_diseases_g** | **DSI_g** | **DPI_g** | **pLI** | **Score_gda** | **EI_gda** | **N_PMIDs** | **N_SNPs_gda** | **First_Ref** | **Last_Ref** |
| --- | --- | --- | --- | --- | --- | --- | --- | --- | --- | --- | --- | --- | --- | --- | --- | --- |
| Diabetic wound | C4728046 | AGER | 177 | Q15109 | advanced glycosylation end-product specific receptor | Receptor | 450 | 0.42 | 0.885 | 6.35E-16 | 0.02 | 1 | 2 | 0 | 2017 | 2018 |
| Diabetic wound | C4728046 | CXCR4 | 7852 | P61073 | C-X-C motif chemokine receptor 4 | G-protein coupled receptor | 739 | 0.362 | 0.923 | 0.017804 | 0.01 | 1 | 1 | 0 | 2017 | 2017 |
| Diabetic wound | C4728046 | PPARG | 5468 | P37231 | peroxisome proliferator activated receptor gamma | Nuclear receptor | 877 | 0.358 | 0.885 | 0.029194 | 0.01 | 1 | 1 | 0 | 2015 | 2015 |
| Diabetic wound | C4728046 | ESR2 | 2100 | Q92731 | estrogen receptor 2 | Nuclear receptor | 528 | 0.4 | 0.923 | 4.45E-08 | 0.01 | 1 | 1 | 0 | 2019 | 2019 |

Table A11. Protein class- kinase

| **Disease** | **Disease_id** | **Gene** | **Gene_id** | **UniProt** | **Gene_Full_Name** | **Protein_Class** | **N_diseases_g** | **DSI_g** | **DPI_g** | **pLI** | **Score_gda** | **EI_gda** | **N_PMIDs** | **N_SNPs_gda** | **First_Ref** | **Last_Ref** |
| --- | --- | --- | --- | --- | --- | --- | --- | --- | --- | --- | --- | --- | --- | --- | --- | --- |
| Diabetic wound | C4728046 | KDR | 3791 | P35968 | kinase insert domain receptor | Kinase | 623 | 0.378 | 0.885 | 0.99982 | 0.03 | 1 | 3 | 0 | 2015 | 2019 |
| Diabetic wound | C4728046 | PIK3CA | 5290 | P42336 | phosphatidylinositol-4,5-bisphosphate 3-kinase catalytic subunit alpha | Kinase | 1511 | 0.292 | 0.923 | 1 | 0.02 | 1 | 2 | 0 | 2019 | 2019 |
| Diabetic wound | C4728046 | PIK3CG | 5294 | P48736 | phosphatidylinositol-4,5-bisphosphate 3-kinase catalytic subunit gamma | Kinase | 1101 | 0.32 | 0.885 | 1.41E-06 | 0.02 | 1 | 2 | 0 | 2019 | 2019 |
| Diabetic wound | C4728046 | PIK3CD | 5293 | O00329 | phosphatidylinositol-4,5-bisphosphate 3-kinase catalytic subunit delta | Kinase | 1119 | 0.319 | 0.885 | 0.99999 | 0.02 | 1 | 2 | 0 | 2019 | 2019 |
| Diabetic wound | C4728046 | PIK3CB | 5291 | P42338 | phosphatidylinositol-4,5-bisphosphate 3-kinase catalytic subunit beta | Kinase | 1083 | 0.322 | 0.885 | 0.99964 | 0.02 | 1 | 2 | 0 | 2019 | 2019 |
| Diabetic wound | C4728046 | MOK | 5891 | Q9UQ07 | MOK protein kinase | Kinase | 251 | 0.476 | 0.769 | 8.84E-22 | 0.01 | 1 | 1 | 0 | 2017 | 2017 |
| Diabetic wound | C4728046 | MAPK3 | 5595 | P27361 | mitogen-activated protein kinase 3 | Kinase | 647 | 0.379 | 0.885 | 0.036884 | 0.01 | 1 | 1 | 0 | 2019 | 2019 |
| Diabetic wound | C4728046 | AKT1 | 207 | P31749 | AKT serine/threonine kinase 1 | Kinase | 1250 | 0.311 | 0.962 | 0.9759 | 0.01 | 1 | 1 | 0 | 2019 | 2019 |

Table A12. Protein class- Enzyme

| **Disease** | **Disease_id** | **Gene** | **Gene_id** | **UniProt** | **Gene_Full_Name** | **Protein_Class** | **N_diseases_g** | **DSI_g** | **DPI_g** | **pLI** | **Score_gda** | **EI_gda** | **N_PMIDs** | **N_SNPs_gda** | **First_Ref** | **Last_Ref** |
| --- | --- | --- | --- | --- | --- | --- | --- | --- | --- | --- | --- | --- | --- | --- | --- | --- |
| Diabetic wound | C4728046 | MMP9 | 4318 | P14780 | matrix metallopeptidase 9 | Enzyme | 1337 | 0.305 | 0.923 | 1.89E-17 | 0.09 | 1 | 9 | 0 | 2017 | 2020 |
| Diabetic wound | C4728046 | NFE2L2 | 4780 | Q16236 | nuclear factor, erythroid 2 like 2 | Enzyme | 823 | 0.357 | 0.885 | 0.003572 | 0.02 | 1 | 2 | 0 | 2016 | 2018 |
| Diabetic wound | C4728046 | MFGE8 | 4240 | Q08431 | milk fat globule-EGF factor 8 protein | Enzyme | 120 | 0.548 | 0.808 | 4.4E-05 | 0.02 | 1 | 2 | 0 | 2016 | 2017 |
| Diabetic wound | C4728046 | MMP8 | 4317 | P22894 | matrix metallopeptidase 8 | Enzyme | 213 | 0.488 | 0.808 | 1.07E-24 | 0.02 | 1 | 2 | 0 | 2018 | 2018 |
| Diabetic wound | C4728046 | PAEP | 5047 | P09466 | progestagen associated endometrial protein | Enzyme | 397 | 0.43 | 0.846 | 3.11E-08 | 0.01 | 1 | 1 | 0 | 2018 | 2018 |
| Diabetic wound | C4728046 | SERPINB3 | 6317 | P29508 | serpin family B member 3 | Enzyme modulator | 101 | 0.565 | 0.615 | 8.21E-16 | 0.01 | 1 | 1 | 0 | 2014 | 2014 |
| Diabetic wound | C4728046 | CDC25C | 995 | P30307 | cell division cycle 25C | Enzyme | 95 | 0.576 | 0.769 | 4.1E-13 | 0.01 | 1 | 1 | 0 | 2017 | 2017 |
| Diabetic wound | C4728046 | DMBT1 | 1755 | Q9UGM3 | deleted in malignant brain tumors 1 | Enzyme | 135 | 0.533 | 0.654 | 4.48E-63 | 0.01 | 1 | 1 | 0 | 2018 | 2018 |
| Diabetic wound | C4728046 | MMP1 | 4312 | P03956 | matrix metallopeptidase 1 | Enzyme | 589 | 0.385 | 0.885 | 7.72E-18 | 0.01 | 1 | 1 | 0 | 2019 | 2019 |
| Diabetic wound | C4728046 | GZMB | 3002 | P10144 | granzyme B | Enzyme | 290 | 0.453 | 0.808 | 3.67E-14 | 0.01 | 1 | 1 | 0 | 2014 | 2014 |

Table A13. Protein class-signaling

| **Disease** | **Disease_id** | **Gene** | **Gene_id** | **UniProt** | **Gene_Full_Name** | **Protein_Class** | **N_diseases_g** | **DSI_g** | **DPI_g** | **pLI** | **Score_gda** | **EI_gda** | **N_PMIDs** | **N_SNPs_gda** | **First_Ref** | **Last_Ref** |
| --- | --- | --- | --- | --- | --- | --- | --- | --- | --- | --- | --- | --- | --- | --- | --- | --- |
| Diabetic wound | C4728046 | VEGFA | 7422 | P15692 | vascular endothelial growth factor A | Signaling | 1899 | 0.266 | 0.923 | 2.41E-05 | 0.05 | 1 | 5 | 0 | 2004 | 2019 |
| Diabetic wound | C4728046 | TGFB1 | 7040 | P01137 | transforming growth factor beta 1 | Signaling | 1558 | 0.287 | 0.962 | 0.03685 | 0.02 | 1 | 2 | 0 | 2003 | 2004 |
| Diabetic wound | C4728046 | VEGFC | 7424 | P49767 | vascular endothelial growth factor C | Signaling | 291 | 0.459 | 0.769 | 0.49565 | 0.01 | 1 | 1 | 0 | 2006 | 2006 |
| Diabetic wound | C4728046 | PGF | 5228 | P49763 | placental growth factor | Signaling | 288 | 0.46 | 0.846 | 0.1288 | 0.01 | 1 | 1 | 0 | 2006 | 2006 |
| Diabetic wound | C4728046 | ANGPTL4 | 51129 | Q9BY76 | angiopoietin like 4 | Signaling | 198 | 0.498 | 0.731 | 2.31E-11 | 0.01 | 1 | 1 | 0 | 2014 | 2014 |
| Diabetic wound | C4728046 | TNF | 7124 | P01375 | tumor necrosis factor | Signaling | 2724 | 0.231 | 0.962 | 0.8033 | 0.01 | 1 | 1 | 0 | 2019 | 2019 |
| Diabetic wound | C4728046 | FN1 | 2335 | P02751 | fibronectin 1 | Signaling | 724 | 0.365 | 0.962 | 0.001405 | 0.01 | 1 | 1 | 0 | 2014 | 2014 |
| Diabetic wound | C4728046 | FGF7 | 2252 | P21781 | fibroblast growth factor 7 | Signaling | 167 | 0.519 | 0.846 | 0.77089 | 0.01 | 1 | 1 | 0 | 2019 | 2019 |
| Diabetic wound | C4728046 | CCN2 | 1490 | P29279 | cellular communication network factor 2 | Signaling | 518 | 0.399 | 0.846 | 0.000502 | 0.01 | 1 | 1 | 0 | 2018 | 2018 |
| Diabetic wound | C4728046 | GPNMB | 10457 | Q14956 | glycoprotein nmb | Signaling | 103 | 0.566 | 0.692 | 2.88E-27 | 0.01 | 1 | 1 | 0 | 2018 | 2018 |
| Diabetic wound | C4728046 | LGALS7 | 3963 | P47929 | galectin 7 | Signaling | 73 | 0.599 | 0.769 | 0.14567 | 0.01 | 1 | 1 | 0 | 2017 | 2017 |
